# Supplementary material for: Tumor cell plasticity, heterogeneity, and resistance in crucial microenvironmental niches in glioma
Source: Nat Commun. 2021 Feb 12;12:1014. doi: 10.1038/s41467-021-21117-3 (PMC7881116; doi:10.1038/s41467-021-21117-3)
Supplement: Supplementary file 1 — Supplementary Information [file 41467_2021_21117_MOESM1_ESM.pdf]

Tumor cell plasticity, heterogeneity and resistance in crucial microenvironmental niches in glioma

Supplementary information

a Uncropped Western blot data

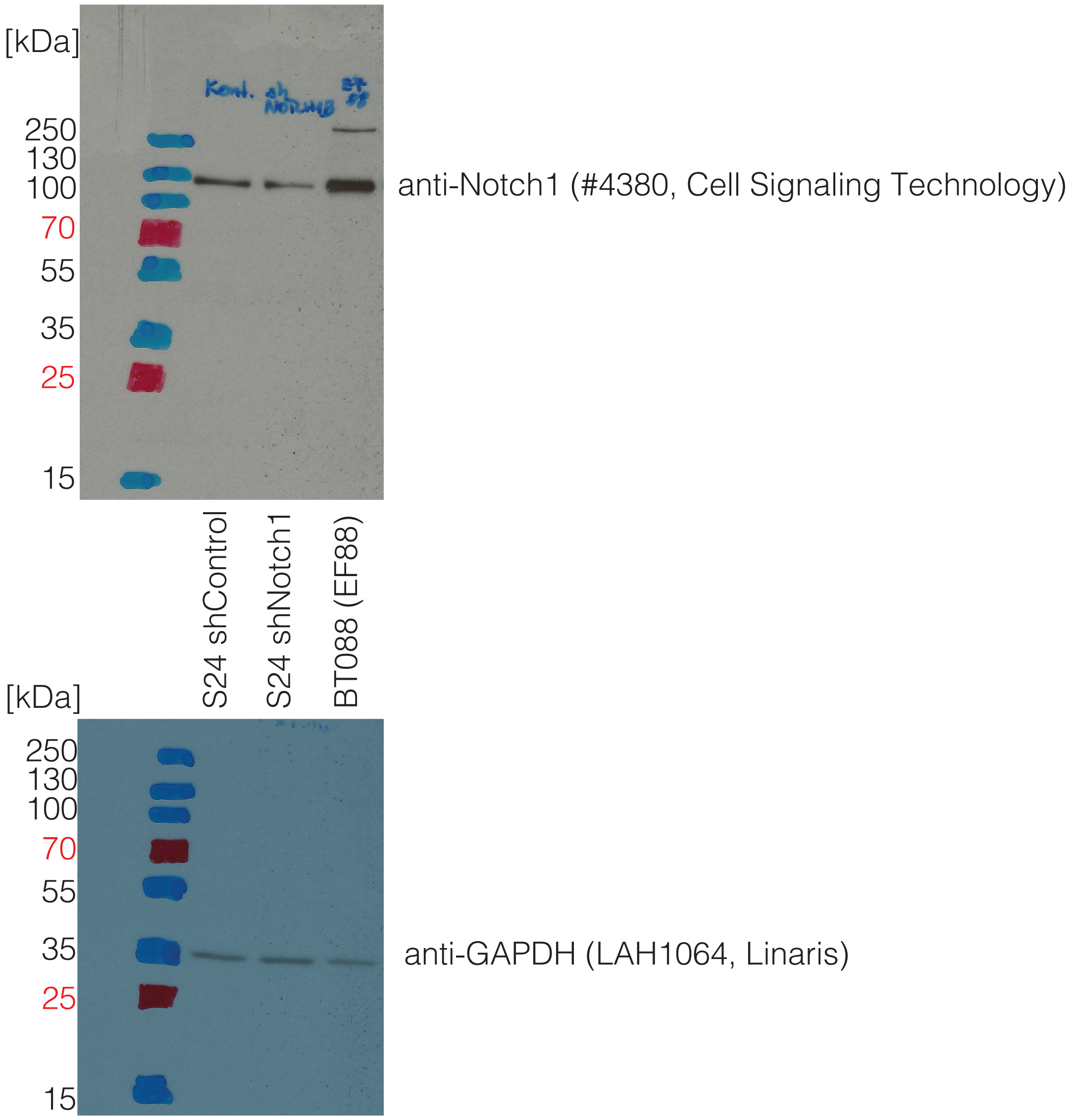

Marker: PageRuler Prestained Protein Ladder (#26619, ThermoFisher Scientific)

b FACS gating strategy

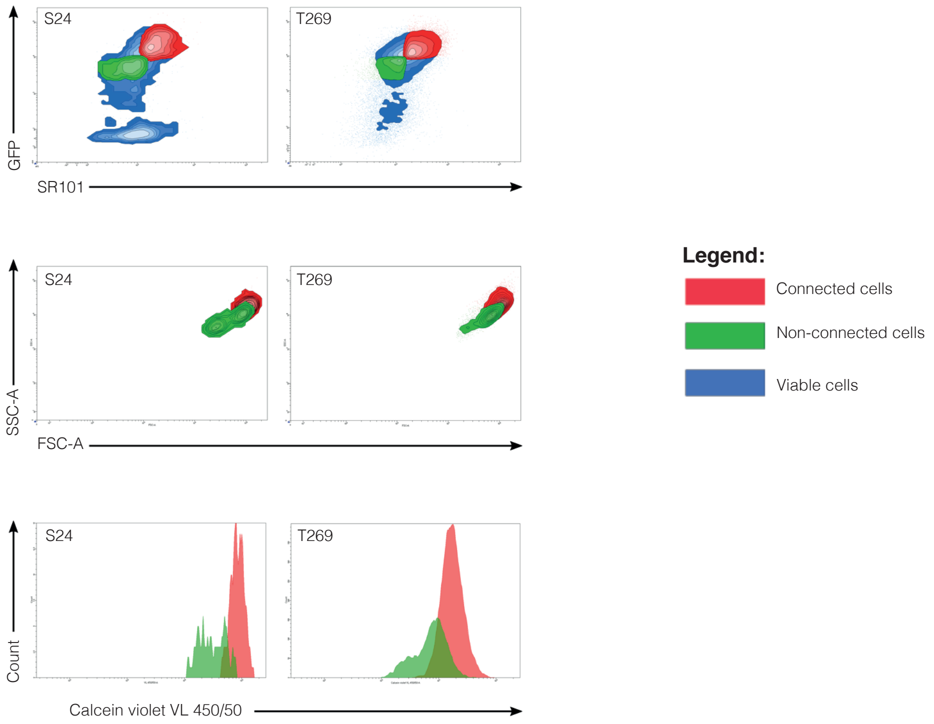

Supplementary Fig. 1

Supplementary Fig. 1. **Uncropped Western Blot data and exemplary FACS gating strategy. a** Uncropped Western blot data of Notch1 expression levels in S24 control, shNOTCH1 and BT088 cells (upper panel). GAPDH loading control (lower panel). Protein ladders and corresponding molecular weights are shown on the left. **b** Exemplary gating strategy for the separation of connected (red) and non-connected (green) tumor cells. Viable cells are shown in blue. FACS was performed as part of another study. The exemplary visualization of the gating strategy was adopted from this study[1].

Supplementary Table 1

Molecular characteristics of primary glioblastoma cell lines

**Name**	**Classifier**	***MGMT***	***IDH1/2***	***CNV***
S24	GB RTK I	methyl.	wild-type	Gain of chr 20, loss of chr 6, 8 and 10, CDKN2A/B del
T269	GB RTK II	methyl.	wild-type	Gain of chr 1, 3, 5, 7, 9, 12-15, 18-20, 22, CDKN2A/B del
T325	No match	unmethyl.	wild-type	Gain of chr 7, loss of chr 4q, 5q, 6, 8, 10 and 11, CDKN2A/B del
P3xx	GB RTK II	methyl.	wild-type	Gain of chr 7, 10p, 19, 20, loss of chr 4, 9, 19q, X, CDKN2A/B del

Adapted from Kessler et al., Cancer Medicine 2020 [2]

Abbreviations:

CNV: copy number variation, LOH: loss of heterozygosity; methyl.: MGMT promoter methylated, unmethyl.: MGMT promoter unmethylated, chr: chromosome, amp: amplification, del: deletion.

Supplementary Table 2

Molecular characteristics of primary oligodendroglioma cell line

**Name**	**Patient histology**	**IDH1/2**	**1p/19q**	***MGMT***
BT088	Anaplastic oligodendroglioma WHO III°	wild-type	LOH	methyl.

Adapted from Kelly et al., Neuro-Oncology 2010 [3]

Abbreviations:

LOH: loss of heterozygosity; methyl.: MGMT promoter methylated.

Supplementary references

1. Xie R, et *al.* Tumor cell network integration in glioma represents a stemness feature. *Neuro Oncol*, (2020).

2. Kessler T*, et al.* Methylome analyses of three glioblastoma cohorts reveal chemotherapy sensitivity markers within DDR genes. *Cancer Med*, (2020).

3. Kelly JJ*, et al.* Oligodendroglioma cell lines containing t(1;19)(q10;p10). *Neuro Oncol* **12**, 745-755 (2010).
