# Supplementary material for: Tumor cell plasticity, heterogeneity, and resistance in crucial microenvironmental niches in glioma
Source: Nat Commun. 2021 Feb 12;12:1014. doi: 10.1038/s41467-021-21117-3 (PMC7881116; doi:10.1038/s41467-021-21117-3)
Supplement: Supplementary file 3 — Reporting Summary [file 41467_2021_21117_MOESM3_ESM.pdf]

nature research

Corresponding author(s): Frank Winkler

Last updated by author(s): Dec 24, 2020

Reporting Summary

Nature Research wishes to improve the reproducibility of the work that we publish. This form provides structure for consistency and transparency in reporting. For further information on Nature Research policies, see our Editorial Policies and the Editorial Policy Checklist.

Statistics

For all statistical analyses, confirm that the following items are present in the figure legend, table legend, main text, or Methods section.

n/a	Confirmed	
☐	☒	The exact sample size (*n*) for each experimental group/condition, given as a discrete number and unit of measurement
☐	☒	A statement on whether measurements were taken from distinct samples or whether the same sample was measured repeatedly
☐	☒	The statistical test(s) used AND whether they are one- or two-sided<br>*Only common tests should be described solely by name; describe more complex techniques in the Methods section.*
☐	☒	A description of all covariates tested
☐	☒	A description of any assumptions or corrections, such as tests of normality and adjustment for multiple comparisons
☐	☒	A full description of the statistical parameters including central tendency (e.g. means) or other basic estimates (e.g. regression coefficient) AND variation (e.g. standard deviation) or associated estimates of uncertainty (e.g. confidence intervals)
☐	☒	For null hypothesis testing, the test statistic (e.g. *F*, *t*, *r*) with confidence intervals, effect sizes, degrees of freedom and *P* value noted<br>*Give P values as exact values whenever suitable.*
☒	☐	For Bayesian analysis, information on the choice of priors and Markov chain Monte Carlo settings
☒	☐	For hierarchical and complex designs, identification of the appropriate level for tests and full reporting of outcomes
☒	☐	Estimates of effect sizes (e.g. Cohen's *d*, Pearson's *r*), indicating how they were calculated

Our web collection on statistics for biologists contains articles on many of the points above.

Software and code

Policy information about availability of computer code

Data collection	In-vivo 2-photon microscopy data were acquired using ZEN Version 14.0.18.201 (Zeiss). FACS analyses were performed with FlowJo (BD Life Sciences) Version 10.5. Confocal microscopy data were acquired using the LAS software Version 2.7.3.9723. RNA sequencing was performed with the commercial software of the HiSeq2000 v4 setup (Illumina, HCS 2.2.58). The quality of bases was evaluated using the FASTX Toolkit. Homertools 4.7 were applied for PolyA-tail trimming; reads with a length of < 17 were removed. The filtered reads were mapped with STAR 2.3 against the human reference genome (GRCh38) and PicardTools 1.78 with CollectRNASeqMetrics were used for quality checking. Count data were generated by htseq-count using the gencode.v26.annotation.gtf file for annotation. DESeq2 1.4.1 was run with default parameters for the group-wise comparison.
Data analysis	All quantifications were performed manually in ImageJ (version 1.53, NIH, RRID:SCR_003070) and Imaris (Bitlane, RRID:SCR_007370). Immunohistochemical and immunofluorescence images were analyzed in Qupath (v0.2.3), Aperio ImageScope software (v11.0.2.725, Aperio Technologies, USA) and ImageJ (version 1.53, NIH, RRID:SCR_003070). Statistical analyses were performed using SigmaPlot (version 14.0, Systat Software, RRID:SCR_003210) and Prism 8.4.1 (GraphPad, RRID:SCR_002798).

For manuscripts utilizing custom algorithms or software that are central to the research but not yet described in published literature, software must be made available to editors and reviewers. We strongly encourage code deposition in a community repository (e.g. GitHub). See the Nature Research guidelines for submitting code & software for further information.

Data

Policy information about availability of data

All manuscripts must include a data availability statement. This statement should provide the following information, where applicable:

- Accession codes, unique identifiers, or web links for publicly available datasets
- A list of figures that have associated raw data
- A description of any restrictions on data availability

Source data are provided with this paper. The RNA seq. data of connected and unconnected S24 and T269 glioblastoma cells have been deposited in the Sequence Read Archive (SRA) database under the accession number PRJNA554870. Uncropped western blot data are provided in Suppl. Fig. 1.

Field-specific reporting

Please select the one below that is the best fit for your research. If you are not sure, read the appropriate sections before making your selection.

[x] Life sciences [ ] Behavioural & social sciences [ ] Ecological, evolutionary & environmental sciences

For a reference copy of the document with all sections, see nature.com/documents/nr-reporting-summary-flat.pdf

Life sciences study design

All studies must disclose on these points even when the disclosure is negative.

Sample size	Sample sizes were estimated based on previous experiments and publications from the group (see Osswald et al., Nature 2014; Jung et al., J Neurosci, 2017; Weil et al. Neurooncology, 2017; Venkataramani et al., Nature, 2019). Following the principles of the 3 R's, several regions were analyzed per animal allowing to obtain comparable levels of data with fewer animals ("reduction").
Data exclusions	No data was excluded from the study.
Replication	All experiments were performed at least in triplicates and measurements were reproducible. Replicates of human tissue stainings and corresponding analyses depended on sample availability.
Randomization	Mice were randomly allocated to the experiments and treatment groups.
Blinding	Acquisition of in vivo data was not performed blinded as tumor cell preparation, implantation and imaging were performed by the same experimenter. Image analyses (including 2-PM, confocal microscopy and MRI images) and histological analyses (human and mice) were performed blinded. Blinding was not relevant for Western blot analyses, as expression levels were normalized to loading controls. Experimenters were blinded for group allocation in survival experiments and analyses.

Reporting for specific materials, systems and methods

We require information from authors about some types of materials, experimental systems and methods used in many studies. Here, indicate whether each material, system or method listed is relevant to your study. If you are not sure if a list item applies to your research, read the appropriate section before selecting a response.

Materials & experimental systems

n/a	Involved in the study	
[ ]	[x]	Antibodies
[ ]	[x]	Eukaryotic cell lines
[x]	[ ]	Palaeontology and archaeology
[ ]	[x]	Animals and other organisms
[ ]	[x]	Human research participants
[x]	[ ]	Clinical data
[x]	[ ]	Dual use research of concern

Methods

n/a	Involved in the study	
[x]	[ ]	ChIP-seq
[ ]	[x]	Flow cytometry
[ ]	[x]	MRI-based neuroimaging

Antibodies

Antibodies used	The following antibodies were used:<br>anti-nestin (ab6320, Abcam, RRID:AB_308832, Lot: GR3293723-1), anti-CD31 (AF3628, R&D Systems, RRID:AB_2161028, Lot: Y2U0119121), anti-aquaporin 4 (ab9512, Abcam, RRID:AB_307299, Lot: GR3270336-1), anti-activated Notch1 (ab8925, Abcam, RRID:AB_306863, Lot: GR218543-60), anti-ki67 (ab15580, Abcam, RRID:AB_443209, Lot: GR3198183-1), anti-nestin (clone 10C2, MAB5326, Merck Millipore, RRID:AB_11211837, Lot: 2697590), anti-IDH1 R132H (DIA-H09, Dianova, RRID:AB_2335716), anti-Ki67 (M7240, Dako, RRID:AB_2142367), anti-CD31 (PA5-16301, ThermoFisher Scientific, RRID: AB_10981955, Lot: VI3068503), anti-Notch1 (#4380, Cell Signaling Technology, RRID:AB_10691684, Lot: 01/2014_2), anti-GAPDH (LAH1064, Linaris), goat anti-rabbit

Alexa Fluor 594 (A-11037, ThermoFisher Scientific, RRID:AB_2534095, Lot: 2160431), goat anti-mouse Alexa Fluor 633 (A-21052, ThermoFisher Scientific, RRID:AB_2535718, Lot: 2126815), donkey anti-mouse IgG Alexa Fluor 488 (A-21202, Thermo Fisher Scientific, RRID:AB_141607, Lot: 2090565), goat anti-mouse IgG Alexa Fluor 488 conjugate (A-11029, Thermo Fisher Scientific, RRID:AB_138404), donkey anti-goat IgG Alexa Fluor 633 (A-21082, Thermo Fisher Scientific, RRID:AB_141493, Lot: 2064015), donkey anti-rabbit IgG Alexa Fluor 546 (A-10040, Thermo Fisher Scientific, RRID:AB_2534016, Lot: 2020130).

Validation

anti-nestin (IHC/IF) (ab6320, Abcam, RRID:AB_308832): a) Jung E, Osswald M, Blaes J, Wiestler B, Sahm F, Schmenger T, Solecki G, Deumelandt K, Kurz FT, Xie R, Weil S, Heil O, Thomé C, Gömmel M, Syed M, Häring P, Huber PE, Heiland S, Platten M, von Deimling A, Wick W, Winkler F. Tweety-Homolog 1 Drives Brain Colonization of Gliomas. J Neurosci. 2017 Jul 19;37(29):6837-6850. doi: 10.1523/JNEUROSCI.3532-16.2017. Epub 2017 Jun 12. PMID: 28607172; PMCID: PMC6705725.
b) https://antibodyregistry.org/search?q=ab6320

anti-CD31 (IHC/IF) (AF3628, R&D Systems, RRID:AB_2161028): a) Romeo SG, Alawi KM, Rodrigues J, Singh A, Kusumbe AP, Ramasamy SK. Endothelial proteolytic activity and interaction with non-resorbing osteoclasts mediate bone elongation. Nat Cell Biol. 2019 Apr;21(4):430-441. doi: 10.1038/s41556-019-0304-7. Epub 2019 Apr 1. PMID: 30936475.
b) https://antibodyregistry.org/search.php?q=AB_2161028

anti-aquaporin 4 (IHC/IF) (ab9512, Abcam, RRID:AB_307299): a) Lee FHF, Zhang H, Jiang A, Zai CC, Liu F. Specific Alterations in Astrocyte Properties via the GluA2-GAPDH Complex Associated with Multiple Sclerosis. Sci Rep. 2018 Aug 27;8(1):12856. doi: 10.1038/s41598-018-31318-4. PMID: 30150703; PMCID: PMC6110783.
b) https://antibodyregistry.org/search.php?q=AB_307299

anti-activated Notch1 (IHC/IF) (ab8925, Abcam, RRID:AB_306863): a) Li H, Ramachandran A, Gao Q, Ravindran S, Song Y, Evans C, George A. Expression and function of NUMB in odontogenesis. Biomed Res Int. 2013;2013:182965. doi: 10.1155/2013/182965. Epub 2013 Jun 6. PMID: 23841055; PMCID: PMC3690219.
b) https://antibodyregistry.org/search.php?q=AB_306863

anti-ki67 (IHC/IF) (ab15580, Abcam, RRID:AB_443209): a) Jung E, Osswald M, Blaes J, Wiestler B, Sahm F, Schmenger T, Solecki G, Deumelandt K, Kurz FT, Xie R, Weil S, Heil O, Thomé C, Gömmel M, Syed M, Häring P, Huber PE, Heiland S, Platten M, von Deimling A, Wick W, Winkler F. Tweety-Homolog 1 Drives Brain Colonization of Gliomas. J Neurosci. 2017 Jul 19;37(29):6837-6850. doi: 10.1523/JNEUROSCI.3532-16.2017. Epub 2017 Jun 12. PMID: 28607172; PMCID: PMC6705725.
b) https://antibodyregistry.org/search.php?q=AB_443209

anti-nestin (IHC) (clone 10C2, MAB5326, Merck Millipore, RRID:AB_11211837): a) Messam CA, Hou J, Berman JW, Major EO. Analysis of the temporal expression of nestin in human fetal brain derived neuronal and glial progenitor cells. Brain Res Dev Brain Res. 2002 Mar 31;134(1-2):87-92. doi: 10.1016/s0165-3806(01)00325-x. PMID: 11947939.
b) https://antibodyregistry.org/search.php?q=AB_11211837

anti-IDH1 R132H (IHC) (DIA-H09, Dianova, RRID:AB_2335716): Capper D, Zentgraf H, Balss J, Hartmann C, von Deimling A. Monoclonal antibody specific for IDH1 R132H mutation. Acta Neuropathol. 2009 Nov;118(5):599-601. doi: 10.1007/s00401-009-0595-z. Epub 2009 Oct 2. PMID: 19798509.
b)https://antibodyregistry.org/search.php?q=AB_2335716

anti-Ki67 (IHC) (M7240, Dako, RRID:AB_2142367): a) Caldera V, Mellai M, Annovazzi L, Piazzi A, Lanotte M, Cassoni P, Schiffer D. Antigenic and Genotypic Similarity between Primary Glioblastomas and Their Derived Neurospheres. J Oncol. 2011;2011:314962. doi: 10.1155/2011/314962. Epub 2011 Aug 18. PMID: 21869887; PMCID: PMC3159011.
b) https://antibodyregistry.org/search?q=AB_2142367

anti-CD31 (IHC) (PA5-16301, ThermoFisher Scientific, RRID: AB_10981955): a) Marusyk A, Tabassum DP, Altrock PM, Almendro V, Michor F, Polyak K. Non-cell-autonomous driving of tumour growth supports sub-clonal heterogeneity. Nature. 2014 Oct 2;514(7520):54-8. doi: 10.1038/nature13556. Epub 2014 Jul 30. PMID: 25079331; PMCID: PMC4184961.
b) https://antibodyregistry.org/search?q=AB_10981955

anti-Notch1 (WB) (#4380, Cell Signaling Technology, RRID:AB_10691684)
https://antibodyregistry.org/search?q=AB_10691684

anti-GAPDH (WB) (LAH1064, Linaris)
https://www.linaris.de/php/zeige.php?id=LAH1064

goat anti-rabbit Alexa Fluor 594 (IHC/IF) (A-11037, ThermoFisher Scientific, RRID:AB_2534095)
https://antibodyregistry.org/search?q=AB_2534095

goat anti-mouse Alexa Fluor 633 (IHC/IF) (A-21052, ThermoFisher Scientific, RRID:AB_2535719)
https://antibodyregistry.org/search?q=AB_2535719

donkey anti-mouse IgG Alexa Fluor 488 (IHC/IF) (A-21202, Thermo Fisher Scientific, RRID:AB_141607)
https://antibodyregistry.org/search?q=AB_141607

goat anti-mouse IgG Alexa Fluor 488 conjugate (IHC/IF) (A-11029, Thermo Fisher Scientific, RRID:AB_138404)
https://antibodyregistry.org/search?q=AB_138404

donkey anti-goat IgG Alexa Fluor 633 (IHC/IF) (A-21082, Thermo Fisher Scientific, RRID:AB_141493)
https://antibodyregistry.org/search?q=AB_141493

donkey anti-rabbit IgG Alexa Fluor 546 (IHC/IF) (A-10040, Thermo Fisher Scientific, RRID:AB_2534016)
https://antibodyregistry.org/search?q=AB_2534016

Eukaryotic cell lines

Policy information about cell lines

Cell line source(s)	Patient-derived glioma cell lines (S24, T269, T325) were established in our laboratory (CCU Neurooncology, German Cancer Research Center, Heidelberg, Germany). The P3xx cell line was provided by H. Miletic (Department of Biomedicine, University of Bergen, Bergen, Norway). BT088 cells (ATCC CRL-3417) were bought from ATCC.
Authentication	S24 (human, female), T269 (human, male), T325 (human, male) and P3xx (human) were authentificated (Multiplexion GmbH, Germany). S24, T269, T325 and P3xx were further authenticated as glioblastoma by Illumina 850k methylation array as described in Venkataramani et al., Nature 2018. BT088 cells were not authenticated. Cell line characteristics can be found in Suppl. Table 1.
Mycoplasma contamination	The cell lines were regularly tested negative for mycoplasma contamination.
Commonly misidentified lines (See ICLAC register)	The study did not involve commonly misidentified cell lines.

Animals and other organisms

Policy information about studies involving animals; ARRIVE guidelines recommended for reporting animal research

Laboratory animals	Male NMRI mice (Charles River and Janvier) with a minimum age of 10 weeks were used.
Wild animals	No wild animals were used.
Field-collected samples	The study did not involve field-collected samples.
Ethics oversight	All animal procedures were performed in accordance with the institutional laboratory animal research guidelines after approval of the responsible animal welfare officer (German Cancer Research Center, Heidelberg, Germany) and the regional council (Referat 35, Regierungspräsidium Karlsruhe, Germany).

Note that full information on the approval of the study protocol must also be provided in the manuscript.

Human research participants

Policy information about studies involving human research participants

Population characteristics	Human research participants varied in age and gender. All patients were adult and treated at the University Hospital Heidelberg or University Hospital Mannheim (University of Heidelberg). Patient samples were anonymised for the study.<br>1. Oligodendroglioma: n=18 (for IDH1 R132H staining), n=16 (for ki67 staining). Methylation class IDH glioma, subclass 1p/19q codeleted oligodendroglioma, all ATRX retained.<br>2. Astrocytoma (IDH1 R132H mutant): n=19 (for IDH1 R132H staining), n=19 (for ki67 staining). Methylation class IDH glioma, subclass astrocytoma, all ATRX loss, all 1p/19q non-codel.<br>3. High grade astrocytoma (IDH1 R132H mutant): n=20 (for IDH1 R132H staining), n=15 (for ki67 staining). Methylation class IDH glioma, subclass high grade astrocytoma, all 1p/19q non-codel. a) IDH1 R132H staining: 16 ATRX loss, 3 ATRX retained, 1 undetermined. b) ki67 staining: 11 ATRX loss, 3 ATRX retained, 1 n/a.<br>4. Glioblastoma (IDH1 wild-type): n=10 (for nestin staining), n=16 (for ki67 staining). Methylation class glioblastoma, IDH wild-type. a) Nestin staining: subclass: RTK I=1, RTK II=7, mesenchymal=1, MYCN=1. All 1p/19q non-codel, all MGMT promoter unmethylated, all TP53 wild-type, 9 ATRX retained, 1 n/a. b) ki67 staining: subclass: RTK I=4, RTK II=9, mesenchymal=3. All 1p/19q non-codel, 6 MGMT promoter methylated, 10 MGMT promoter unmethylated.
Recruitment	Samples were obtained from the Department of Neuropathology after surgical resection of glioma at the University Hospital Heidelberg or the University Hospital Mannheim (University of Heidelberg). All patients gave their informed consent for the scientific use of resected tissue samples. Samples were selected for blinded immunohistochemical analyses based on their molecular features ruling out biases such as self-selection bias.
Ethics oversight	Ethics committee, Heidelberg University, Heidelberg, Germany (Ethikkommission der Universität Heidelberg)

Note that full information on the approval of the study protocol must also be provided in the manuscript.

Flow Cytometry

Plots

Confirm that:

- [ ] The axis labels state the marker and fluorochrome used (e.g. CD4-FITC).
- [ ] The axis scales are clearly visible. Include numbers along axes only for bottom left plot of group (a 'group' is an analysis of identical markers).
- [ ] All plots are contour plots with outliers or pseudocolor plots.
- [ ] A numerical value for number of cells or percentage (with statistics) is provided.

Methodology

Sample preparation	Whole brain single cell suspensions were prepared with brain tumor dissociation kit (130-095-942, Miltenyi Biotec) and gentleMACSTM Dissociator (Miltenyi Biotec). After dissociation the suspension was resuspended in FACS buffer (PBS+ 1% FCS, 10500064, ThermoFisher). Cells were stained with Calcein Violet 450 AM (65-0854-39, Invitrogen) and TO-PRO®-3 Iodide (T3605, Invitrogen) for 10 min on ice before sorting for the viable cell population (Calcein Violet 450high and TO-PRO-3neg). Within the viable cell population SR101high, GFPhigh (connected tumor cells) and SR101low, GFPlow (unconnected tumor cells) were separated. The YG586/15 channel was used to visualize SR101 signal.
Instrument	FACSAria cell sorter (BD Biosystems)
Software	Data was collected with the FACS Diva Software (v9, BD Biosciences). Data was analysed with FlowJo (v10.5) (BD Life Sciences)
Cell population abundance	GFP-positive S24 GBMSCs were >95% postive post sort.
Gating strategy	Boundaries between positive and negative cells was defined by FMO (fluorescence minus one).

- [x] Tick this box to confirm that a figure exemplifying the gating strategy is provided in the Supplementary Information.

Magnetic resonance imaging

Experimental design

Design type	n/a (no functional/fMRI measurements)
Design specifications	n/a (no functional/fMRI measurements)
Behavioral performance measures	n/a (no functional/fMRI measurements)

Acquisition

Imaging type(s)	structural
Field strength	9.4T
Sequence & imaging parameters	T2-weighted rapid acquisition with refocused echoes (RARE) sequence, parameters: TE = 33 ms, TR = 2500 ms, flip angle = 90°, acquisition matrix: 200 x 150, number of averages = 2, slice thickness = 700 µm, duration = 2 min 53 s
Area of acquisition	whole brain neuroimaging

Diffusion MRI [ ] Used [x] Not used

Preprocessing

Preprocessing software	n/a (no preprocessing was performed)
Normalization	n/a (no normalization was performed)
Normalization template	n/a (no normalization was performed)
Noise and artifact removal	n/a (no noise or artifact removal was performed)
Volume censoring	n/a (no volume censoring was performed)

Statistical modeling & inference

Model type and settings	n/a (no statistical modeling was performed)

Effect(s) tested	n/a (no effects were tested)

Specify type of analysis: [x] Whole brain [ ] ROI-based [ ] Both

Statistic type for inference (See Eklund et al. 2016)	n/a (no interference)
Correction	n/a (no correction was performed)

Models & analysis

n/a	Involved in the study
[x]	[ ] Functional and/or effective connectivity
[x]	[ ] Graph analysis
[x]	[ ] Multivariate modeling or predictive analysis
